# Myocardial injury in hospitalized COVID-19 patients: a retrospective study, systematic review, and meta-analysis

**DOI:** 10.1186/s12872-021-02450-3

**Published:** 2021-12-31

**Authors:** Khalid Changal, Spiro Veria, Sean Mack, David Paternite, Shoaib Altaf Sheikh, Mitra Patel, Tanveer Mir, Mujeeb Sheikh, P. Kasi Ramanathan

**Affiliations:** 1 Cardiovascular Medicine, University of Toledo, Toledo, OH USA; 2 Department of Medicine, University of Toledo College of Medicine and Life Sciences, Toledo, OH USA; 3 Internal Medicine, Nazareth Hospital (Trinity Health), Philadelphia, PA USA; 4 Internal Medicine, Detroit Medical Center, Wayne State University, Detroit, MI USA; 5 Department of Cardiovascular Medicine, Interventional Cardiology, Promedica Toledo Hospital, 2109 Hughes Dr, Jobst Tower 3rd Floor, Toledo, OH 43606 USA

## Abstract

**Introduction:**

The majority of studies evaluating the effect of myocardial injury on the survival of COVID-19 patients have been performed outside of the United States (U.S.). These studies have often utilized definitions of myocardial injury that are not guideline-based and thus, not applicable to the U.S. patient population.

**Methods:**

The current study is a two-part investigation of the effect of myocardial injury on the clinical outcome of patients hospitalized with COVID-19. The first part is a retrospective analysis of 268 patients admitted to our healthcare system in Toledo, Ohio, U.S.; the second part is a systematic review and meta-analysis of all similar studies performed within the U.S.

**Results:**

In our retrospective analysis, patients with myocardial injury were older (mean age 73 vs. 59 years, *P* 0.001), more likely to have hypertension (86% vs. 67%, *P* 0.005), underlying cardiovascular disease (57% vs. 24%, *P* 0.001), and chronic kidney disease (26% vs. 10%, *P* 0.004). Myocardial injury was also associated with a lower likelihood of discharge to home (35% vs. 69%, *P* 0.001), and a higher likelihood of death (33% vs. 10%, *P* 0.001), acute kidney injury (74% vs. 30%, *P* 0.001), and circulatory shock (33% vs. 12%, *P* 0.001). Our meta-analysis included 12,577 patients from 8 U.S. states and 55 hospitals who were hospitalized with COVID-19, with the finding that myocardial injury was significantly associated with increased mortality (HR 2.43, CI 2.28–3.6, *P* 0.0005). The prevalence of myocardial injury ranged from 9.2 to 51%, with a mean prevalence of 27.2%.

**Conclusion:**

Hospitalized COVID-19 patients in the U.S. have a high prevalence of myocardial injury, which was associated with poorer survival and outcomes.

**Supplementary Information:**

The online version contains supplementary material available at 10.1186/s12872-021-02450-3.

## Introduction

The COVID-19 pandemic continues to affect millions of people in the United States (U.S.) and across the world [1]. Myocardial injury, as reflected by an increase in the serum troponin level above the 99th percentile, has been found to be relatively common in hospitalized COVID-19 patients and may predict a poor prognosis. However, most of these studies have been performed outside of the U.S. [2–4] and utilize definitions of myocardial injury that are not guideline-based, thus making it difficult to apply such findings to the hospitalized COVID-19 population within the U.S. [5–7]. The goal of the present study is to determine the prevalence of myocardial injury in the hospitalized COVID-19 patient population in the U.S., compare outcomes with hospitalized patients who have COVID-19 without myocardial injury, and to determine the risk factors for the development of myocardial injury and possible differences in clinical outcome between the two groups. In addition, we carry out a systematic review of current U.S. studies, summarize their findings, and perform a meta-analysis.

## Methods

This study has two parts: one is a retrospective analysis of patients admitted to our health system; the second is a systematic review and meta-analysis of all similar studies performed in the U.S.

We conducted a retrospective study of a hospitalized patient population at two tertiary care hospitals in Toledo, Ohio, U.S. Adult (> 18 years) patients who were hospitalized with the diagnosis of COVID-19 from 1 January 2020 through 1 May 2020 were included. Patients with type 1, 3, 4, and 5 myocardial infarctions were excluded from the study. Real-Time RT-PCR (cobas® SARS-CoV-2 Test) was used to establish COVID-19 diagnosis via nasopharyngeal and oropharyngeal swab samples obtained from our patients.

Myocardial injury was defined using the fourth universal definition of myocardial infarction as below:

Detection of a rise and/or fall of cTn with at least one value above the 99th percentile (in our laboratory this a troponin I > 0.04 ng/ml) and myocardial oxygen supply and demand mismatch not related to coronary thrombosis, with a minimum of one of the following: clinical symptoms related to cardiac ischemia; electrocardiographic changes suggestive of ischemia; new and pathological Q waves; imaging suggestive of acute loss of viable myocardium, or incident regional wall motion abnormalities consistent with ischemia [8].

All hospitalized COVID-19 patients received an EKG on admission. The present study only included patients who had serum troponin levels checked, which was based on clinical suspicion and/or EKG or imaging abnormalities. Eleven patients were excluded from the study as serum troponin was not measured in their cases.

Data were collected by review of individual electronic medical records from the hospital database. Data were collected on demographics, baseline comorbidities, hospital course, clinical and laboratory variables, cost, and outcomes. The underlying cardiovascular disease (CVD) status was identified by review of patient charts. To qualify for the CVD group, patients needed to have at least one of the following diagnoses: ischemic heart disease, congestive heart failure, and/or atrial fibrillation.

The statistics were performed using Statistical Package for the Social Sciences (SPSS), Version 20.0. We used mean, standard deviation/standard error of mean, and percentage when appropriate for the patient’s characteristic description. Group differences were compared using the Pearson *χ*^2^ or Fisher's exact test for categorical variables, or the Student *t* test for continuous variables. *P* values < 0.05 were regarded as significant. We conducted univariate and multivariate regression analysis for factors contributing to myocardial injury, and for the effect of myocardial injury on different outcomes. The variables with statistical significance on univariate analysis were analyzed with multivariate analysis. Multivariate analysis was performed separately for clinical factors (4 variables) and for hospital course/clinical outcomes (4 variables).

The Institutional Review Board of Promedica Health System in Toledo, Ohio, U.S. approved the current study.

Review and meta-analysis were performed using Preferred Reporting Items for Systematic Reviews and Meta-Analyses guidelines. The study methodology is described in the Additional file 1. We exclusively included studies performed in the U.S. We searched multiple databases using keywords such as “myocardial injury”, “troponin”, and “COVID-19”. A systematic review was performed. Quantitative meta-analysis was performed only on studies that had clear outcomes defined. One study was excluded as it included only pregnant patients. We used hazard ratio (HR) and corresponding 95% confidence interval (CI) for measuring mortality outcomes. Meta-analysis was performed using Review Manager Version 5.3 (The Nordic Cochrane Center, The Cochrane Collaboration, 2014), R version 3.6.2.

## Results

A total of 268 patients were included in this study at our health system in Toledo, Ohio, U.S. Fifty-eight (22.4%) patients met the inclusion criteria of myocardial injury, and 210 patients were included in the no myocardial injury group. Baseline characteristics are described in Table 1. Patients with myocardial injury were older (mean age 73 vs. 59 years, *P* 0.001), more likely to have hypertension (86% vs. 67%, P 0.005), underlying cardiovascular disease (57% vs. 24%, *P* 0.001), ischemic heart disease (35% vs. 16%, *P* 0.003), heart failure with reduced ejection fraction (12% vs. 3%, *P* 0.009), heart failure with preserved ejection fraction (19% vs. 7%, *P* 0.012), atrial fibrillation (21% vs. 7%, *P* 0.005), history of stroke (28% vs. 8%, *P* 0.001), chronic kidney disease (26% vs. 10%, *P* 0.004), and end-stage renal disease (12% vs. 2%, *P* 0.003). No significant statistical difference was noticed for sex, race, history of diabetes mellitus, active cancer, chronic liver disease, or home ACE inhibitor or Angiotensin II receptor blocker therapy. Patients in the myocardial injury group were less likely to initially present with fever (40% vs. 58%, *P* 0.022), dry cough (47% vs. 68%, *P* 0.005), or myalgias (9% vs. 27%, *P* 0.006). Serum troponins were measured in 97% of patients hospitalized for COVID-19.

**Table 1:** Patient characteristics and comorbidities; comparison of patients with and without myocardial injury

Baseline characteristics	All patients (N = 268)	Myocardial injury (N = 58)	No myocardial injury (N = 210)	*P* value
Age	62 ± 17	73 ± 14	59 ± 17	< 0.001
Sex				
Male, n (%)	139 (52)	30 (52)	109 (52)	1.000
Female, n (%)	129 (48)	28 (48)	101 (48)	
Race				
Caucasian, n (%)	172 (64)	43 (74)	129 (62)	0.176
African–American, n (%)	80 (30)	13 (22)	67 (32)	
Latino, n (%)	13 (5)	1 (1)	12 (6)	
Other, n (%)	2 (1)	1 (1)	1 (1)	
Hypertension, n (%)	191 (71)	50 (86)	141 (67)	0.005
Diabetes mellitus, n (%)	97 (36)	24 (41)	73 (35)	0.359
Cardiovascular disease, n (%)	84 (31)	33 (57)	51 (24)	< 0.001
Ischemic heart disease, n (%)	53 (20)	20 (35)	33 (16)	0.003
HFrEF, n (%)	13 (5)	7 (12)	6 (3)	0.009
HFpEF, n (%)	26 (10)	11 (19)	15 (7)	0.012
Atrial fibrillation, n (%)	27 (10)	12 (21)	15 (7)	0.005
Active cancer, n (%)	11 (4)	1 (2)	10 (5)	0.466
Stroke, n (%)	33 (12)	16 (28)	17 (8)	< 0.001
Chronic kidney disease, n (%)	36 (13)	15 (26)	21 (10)	0.004
ESRD on HD, n (%)	11 (4)	7 (12)	4 (2)	0.003
Chronic liver disease, n (%)	13 (5)	3 (5)	10 (5)	1.000
Immunosuppressive state, n (%)	17 (6)	7 (12)	10 (5)	0.064
Home med: ACEi, n (%)	60 (23)	15 (26)	45 (22)	0.297
Home med: ARBs/ARNI, n (%)	31 (12)	5 (9)	26 (12)	0.496
Presenting vital signs				
Heart rate	93 ± 19	92 ± 23	93 ± 17	0.686
Respiratory rate	22 ± 6	24 ± 8	22 ± 6	0.104
Systolic blood pressure	127 ± 21	123 ± 22	128 ± 21	0.158
Reasons for hospitalization				
Chest pain, n (%)	49 (18)	7 (12)	42 (20)	0.185
Palpitations, n (%)	4 (2)	2 (4)	2 (1)	0.192
Fever, n (%)	142 (54)	22 (40)	120 (58)	0.022
Malaise, n (%)	120 (46)	21 (38)	99 (48)	0.224
Dry Cough, n (%)	167 (64)	26 (47)	141 (68)	0.005
Anorexia, n (%)	39 (15)	4 (7)	35 (17)	0.089
Myalgia, n (%)	60 (23)	5 (9)	55 (27)	0.006
Dyspnea, n (%)	215 (82)	44 (80)	171 (83)	0.690
Orthopnea, n (%)	1 (0.4)	0 (0)	1 (0.5)	1.000
Expectoration, n (%)	24 (9)	4 (7)	20 (10)	0.794
Diarrhea, n (%)	66 (25)	8 (15)	58 (28)	0.054
Headache, n (%)	40 (15)	4 (7)	36 (18)	0.090
Vomiting, n (%)	37 (14)	6 (11)	31 (15)	0.519
Abdominal pain, n (%)	23 (9)	6 (11)	17 (8)	0.592

*HFpEF* congestive heart failure with preserved ejection fraction, EF < 40%, *HFrEF* congestive heart failure with reduced ejection fraction, EF > 50%. There were 3 patients with HfmrEF (EF 40–50%) and were included in HfrEF group. ESRD on HD = End stage renal disease on hemodialysis. *Immunosuppressive state *anyone on chronic immunomodulatory drugs or with immunodeficiencies such as HIV, *ARNI* angiotensin receptor-neprilysin inhibitor, *EKG* electrocardiogram

Table 2 describes the effect of myocardial injury on hospital course and clinical outcomes. Patients with myocardial injury were more likely to have QT prolongation (36% vs. 21%, *P* 0.025), with longer QTc measurements (457 ms vs. 443 ms, *P* 0.008), and abnormal EKG readings (60% vs. 30%, *P* 0.001), with greater likelihood of having arrhythmias (28% vs. 7%, *P* 0.001), atrial fibrillation (19% vs. 6%, *P* 0.007), ST depression or T wave inversion (22% vs. 11%, *P* 0.031). Patients with myocardial injury were also more likely to have abnormal BNP (56% vs. 23%, *P* 0.001), elevated D-dimer (84% vs. 69%, *P* 0.028) with higher D-dimer peak (5854 vs. 2640, *P* 0.022), acute kidney injury (74% vs. 30%, *P* 0.001), and circulatory shock (33% vs. 12%, *P* 0.001). Patients with myocardial injury had a lower likelihood of discharge to home (35% vs. 69%, *P* 0.001) and a higher likelihood of death (33% vs. 10%, *P* 0.001). Patients with myocardial injury were more likely to have received therapeutic anticoagulation (18% vs. 6%, *P* 0.025), steroid therapy (32% vs. 11%, *P* 0.001), and less likely to have received angiotensin II receptor blocker therapy during their hospital course (3% vs. 14%, *P* 0.035). There was no association between myocardial injury and length of stay or cost of hospitalization.

**Table 2:** Patient outcomes and laboratory studies during hospitalization, and comparison of patients with and without myocardial injury

Clinical course/outcome	All patients (N = 268)	Myocardial injury (N = 58)	No myocardial injury (N = 210)	*P* value
Longest QTc measurement (ms)	446 ± 37	457 ± 37	443 ± 36	0.008
Cardiac arrest, n (%)	1 (0.4)	0	1 (0.5)	1.000
EKG and cardiac rhythm abnormalities				
Arrhythmia, n (%)	30 (11)	16 (28)	14 (7)	< 0.001
Atrial fibrillation, n (%)	24 (9)	11 (19)	13 (6)	0.007
Sustained VT, n (%)	3 (1)	2 (3)	1 (0.5)	0.119
VT, n (%)	4 (1.5)	2 (3)	2 (1)	0.205
1st degree heart block, n (%)	6 (2)	2 (3)	4 (2)	0.613
2nd (Type 2) or 3rd degree heart block, n (%)	2 (1)	0 (0)	2 (1)	1.000
New left or right bundle branch block, n (%)	23 (9)	4 (7)	19 (9)	0.793
ST depression or T wave inversion, n (%)	36 (14)	13 (22)	23 (11)	0.031
ST elevation, n (%)	5 (2)	3 (5)	2 (1)	0.069
QT prolongation	66 (25)	21 (36)	45 (21)	0.025
Abnormal EKG, n (%)	99 (37)	35 (60)	64 (30)	< 0.001
Any arrhythmia, n (%)	39 (15)	17 (29)	22 (11)	0.001
Troponin I peak (ng/mL)	0.34 ± 1.50	1.48 ± 2.97	0.02 ± 0.02	< 0.001
Abnormal BNP, n (%)	45 (30)	22 (56)	23 (21)	< 0.001
BNP peak (pg/mL)	185 ± 299	373 ± 411	189 ± 22	< 0.001
High d-dimer, n (%)	184 (72)	47 (84)	137 (69)	0.028
D-dimer peak (ng/mL)	3254 ± 8868	5854 ± 12,899	2640 ± 7482	0.022
Acute kidney injury, n (%)	107 (40)	43 (74)	64 (30)	< 0.001
Peak creatinine (mg/dL)	1.85 ± 2.15	3.10 ± 3.46	1.51 ± 1.49	< 0.001
New HD or CVVHD, n (%)	6 (2)	1 (2)	5 (2)	1.000
Invasive ventilation, n (%)	50 (19)	13 (22)	37 (18)	0.447
Shock of any type, n (%)	44 (16)	19 (33)	25 (12)	< 0.001
ARDS, n (%)	41 (15)	11 (19)	30 (14)	0.407
Ischemic Stroke, n (%)	2 (1)	1 (2)	1 (0.5)	0.382
DVT and/or PE, n (%)	10 (4)	4 (7)	6 (3)	0.228
Death, n (%)	41 (15)	19 (33)	22 (10)	< 0.001
Discharge				
Home, n (%)	165 (62)	20 (35)	145 (69)	< 0.001
SNF, n (%)	59 (22)	17 (29)	42 (20)	
LOS (days)	9 ± 9	9 ± 10	9 ± 9	0.866
Cost of hospitalization (US dollars)	92,727 ± 125,821	84,271 ± 104,119	95,535 ± 131,126	0.547
In hospital medications				
Hydroxychloroquine, n (%)	187 (70)	37 (65)	150 (72)	0.329
Azithromycin, n (%)	40 (15)	10 (18)	30 (14)	0.536
Hydroxychloroquine AND Azithromycin, n (%)	33 (12)	8 (14)	25 (12)	0.654
Tocilizumab, n (%)	6 (2)	0 (0)	6 (3)	0.346
> 1 QT prolonging drug, n (%)	139 (52)	30 (53)	109 (52)	1.000
Therapeutic anticoagulation, n (%)	23 (9)	10 (18)	13 (6)	0.025
Steroids, n (%)	41 (15)	18 (32)	23 (11)	< 0.001
ACEi/ARNI, n (%)	40 (15)	5 (9)	35 (17)	0.149
ARBs, n (%)	31 (12)	2 (3)	29 (14)	0.035

Abnormal D-dimer was defined by more than the lab specified value of 255 ng/mL. High troponin was defined by a value more than the lab specified value of 0.04 ng/mL. QTc was considered prolonged if more than 460 ms in men and more than 480 ms in women on any EKG done during hospital stay

SI units for BNP = pg/mL Abnormal BNP was defined by a value of more than 100 pg/mL

Units for creatinine = mg/dL

SI units for troponin I = ng/mL

*AMA* against medical advice, *ARNI* angiotensin receptor-neprilysin inhibitor, *VT* ventricular tachycardia, *ARDS* acute respiratory distress syndrome, *PE* pulmonary embolism, *HD* hemodialysis, *CVVD* continuous venovenous hemodialysis, *SNF* skilled nursing facility, *LOS* length of stay

Univariate and Multivariate regression analysis were performed as detailed in Table 3. On univariate analysis, the odds of having myocardial injury were higher with age (OR 1.06, 95% CI 1.04–1.08), hypertension (OR 3.06, 95% CI 1.37–6.81), underlying cardiovascular disease (OR 4.12, 95% CI 2.24–7.56), ischemic heart disease (OR 2.82, 95% CI 1.46–5.44), congestive heart failure with reduced ejection fraction (OR 4.67, 95% CI 1.50–14.49), congestive heart failure with preserved ejection fraction (OR 3.04, 95% CI 1.31–7.05), atrial fibrillation (OR 3.39, 95% CI 1.49–7.73), and end stage renal disease (OR 7.07, 95% CI 1.99–25.07). The odds of having myocardial injury were also higher with abnormal EKG (OR 3.47, 95% CI 1.90–6.34), and EKG findings of arrhythmia (OR 5.46, 95% CI 2.47–12.07), atrial fibrillation (OR 3.53, 95% CI 1.49–8.37), ST depression or T wave inversion (OR 2.34, 95% CI 1.09–4.97), and QT prolongation (OR 2.08, 95% CI 1.11–3.90). Additionally, the odds of having myocardial injury were also higher with abnormal BNP (OR 4.95, 95% CI 2.27–10.82), high D-dimer (OR 2.40, 95% CI 1.11–5.20), acute kidney injury (OR 3.39, 95% CI 3.39–12.62), shock of any type (OR 3.70, 95% CI 1.85–7.39), do not resuscitate & comfort care status (OR 4.66, 95% CI 2.49–8.71), discharge to skilled nursing facility (OR 2.94, 95% CI 1.41–6.10), and death (OR 4.25, 95% CI 2.09–8.61). On multivariate analysis, the association of myocardial injury with age, underlying cardiovascular disease, end stage renal disease, arrhythmia on EKG, abnormal BNP, and acute kidney injury was confirmed.

**Table 3:** Univariate and multivariate analysis for factors associated with myocardial injury

Clinical factors	Univariate analysis			Multivariate analysis		
	Odds ratio	95% confidence interval	*P* value	Odds ratio	95% confidence interval	*P* value
Age (years)	1.06	1.04–1.08	< 0.001	1.05	1.02–1.1	0.00
Sex	1.01	0.56–1.80	0.981	–	–	–
Hypertension	3.06	1.37–6.81	0.006	1.37	0.56–3.36	0.48
Diabetes mellitus	1.33	0.73–2.40	0.354	–	–	–
Cardiovascular disease	4.12	2.24–7.56	< 0.001	2.0	1.1–4.0	0.04
Ischemic heart disease	2.82	1.46–5.44	0.002	–	–	–
HFrEF	4.67	1.50–14.49	0.008	–	–	–
HFpEF	3.04	1.31–7.05	0.009	–	–	–
Atrial fibrillation	3.39	1.49–7.73	0.004	–	–	–
ESRD on HD	7.07	1.99–25.07	0.002	6.62	1.7–25	0.06
*Hospital course and clinical outcome*						
EKG findings						
Arrhythmia	5.46	2.47–12.07	< 0.001	3.04	1.1–9.3	0.04
Atrial fibrillation	3.53	1.49–8.37	0.004	–	–	–
1st degree heart block	1.84	0.33–10.30	0.488	–	–	–
New BBB	0.75	0.24–2.28	0.606	–	–	–
ST depression or T wave inversion	2.34	1.09–4.97	0.027	–	–	–
QT prolongation	2.08	1.11–3.90	0.022	–	–	–
Abnormal EKG	3.47	1.90–6.34	< 0.001	–	–	–
Abnormal BNP	4.95	2.27–10.82	< 0.001	3.03	1.3–7.2	0.01
High d-dimer	2.40	1.11–5.20	0.026	1.2	0.4–3.5	0.73
Acute kidney injury	6.54	3.39–12.62	< 0.001	4.5	1.8–11	0.001
Invasive ventilation	1.35	0.66–2.75	0.408	–	–	–
Shock of any type	3.70	1.85–7.39	< 0.001	–	–	–
ARDS	1.44	0.67–3.08	0.354	–	–	–
Ischemic stroke	3.73	0.23–60.61	0.354	–	–	–
Length of stay	0.99	0.96–1.03	0.865	–	–	–
Do not resuscitate and Comfort care	4.66	2.49–8.71	< 0.001	–	–	–
Discharge to skilled Nursing facility	2.94	1.41–6.10	0.004	–	–	–
Death	4.25	2.09–8.61	< 0.001	–	–	–
In hospital medications						
Hydroxychloroquine	0.73	0.39–1.36	0.316	–	–	–
Anticoagulation	1.76	1.01–3.06	0.046	–	–	–
ACEi/ARNi	0.47	0.18–1.27	0.14	–	–	–
Steroids	3.73	1.84–7.57	< 0.001	–	–	–

*HFpEF* congestive heart failure with preserved ejection fraction, *HFrEF* congestive heart failure with reduced ejection, *ARNI* angiotensin receptor-neprilysin inhibitor, *ESRD on HD* End stage renal disease on hemodialysis, *BBB* bundle branch block (complete left or right). OR for continuous variables is calculated for 1-unit increments

### Systematic review and meta-analysis

Including the current study, a total of 7 studies were included in the review (Table 4) [8–13]. Six studies were included in quantitative analysis. All studies were retrospective in design and included only hospitalized COVID-19 patients within the U.S. The study by Pachtman et al*.* [13] was excluded as it included only pregnant patients. The quantitative analysis included data from 8 states or 55 hospitals. 12,577 patients were included in the quantitative analysis. The prevalence of myocardial injury ranged from 9.2 to 51%. The overall mean prevalence of myocardial injury was 27.2%. All studies included hospitalized patients.

**Table 4:** Details of studies included in systematic review and meta-analysis

References, study design	State, health system, number of hospitals	Total number of patients	Myocardial Injury N (%)	No myocardial injury N (%)	Troponin assay used	Patient population	Main finding
Lala et al. [10], Retrospective	New York, Mount Sinai Health System, 5	2736	985 (36)	1751 (64)	Cardiac troponin I (Abbott Architect)	Hospitalized	COVID-19 patients with CVD were more likely to have myocardial injury than patients without CVD. Troponin elevation among patients hospitalized with COVID-19 was associated with higher risk of mortality
Majure et al. [11] Retrospective	New York, Northwell Health System 13	6247	1821 (29)	4426 (71)	cardiac troponin I (Siemens Dimension Vista; Siemens Dimension EXL Systems); cardiac troponin T (Roche Troponin T STAT, 4th generation; Elecys Troponin T Gen 5 STAT)	Hospitalized	Patients hospitalized with COVID-19 and elevated troponin had increased mortality compared with patients with normal troponin levels, which was independent of cardiovascular co-morbidities and elevated inflammatory markers
Case et al. [12] Retrospective	Maryland (& Washington D.C.) MedStar Health System 11	2716	250 (9.2)	2466 (90.8)	Troponin I	Hospitalized	COVID-19 patients with troponin elevation were at higher risk for mechanical ventilation and in-hospital mortality
De Michieli et al. [13] Retrospective	Minnesota, Wisconsin, Florida, Arizona Mayo Clinic Health System 17	367	169 (46)	198 (54)	Hs-cardiac troponin T (Elecys Troponin T Gen 5 STAT)	Hospitalized	Myocardial injury is prognostic in COVID-19 patients with regard to short-term mortality and major adverse events. A single hs-cTnT < 6 ng/L at presentation was associated with a more favorable prognosis
Pachtman Shetty et al. [14] Retrospective	New York Northwell Health 7	18	4 (22)	14 (78)	Hs-Trop, Troponin T, Troponin I	Pregnant and immediately postpartum hospitalized for COVID-19	Among pregnant women hospitalized for COVID-19, 20% were found to have elevations in troponin and 30% had elevated BNP
Metkus et al. [9] Retrospective	Maryland, Johns Hopkins Health System 5	243	124 (51)	119 (49)	Troponin T or Troponin I	COVID-19 patients who required intubation	Myocardial injury in patients with severe COVID-19 was a function of comorbidities, age, and multisystem organ dysfunction Myocardial injury was associated with > twofold hazard for death
Changal (2021) Retrospective*	Ohio, Promedica Health System 2	258	58 (22.5)	210 (77.5)	Troponin I (Sunquest)	Hospitalized COVID-19 patients	Myocardial injury was common, and predicted mortality, poor outcomes, and discharge to skilled nursing facility

*Our study as detailed in this manuscript

*CVD* cardiovascular disease, *hs-cTnT* high sensitivity cardiac troponin-t, *BNP* brain natriuretic peptide

**Fig. 1:**
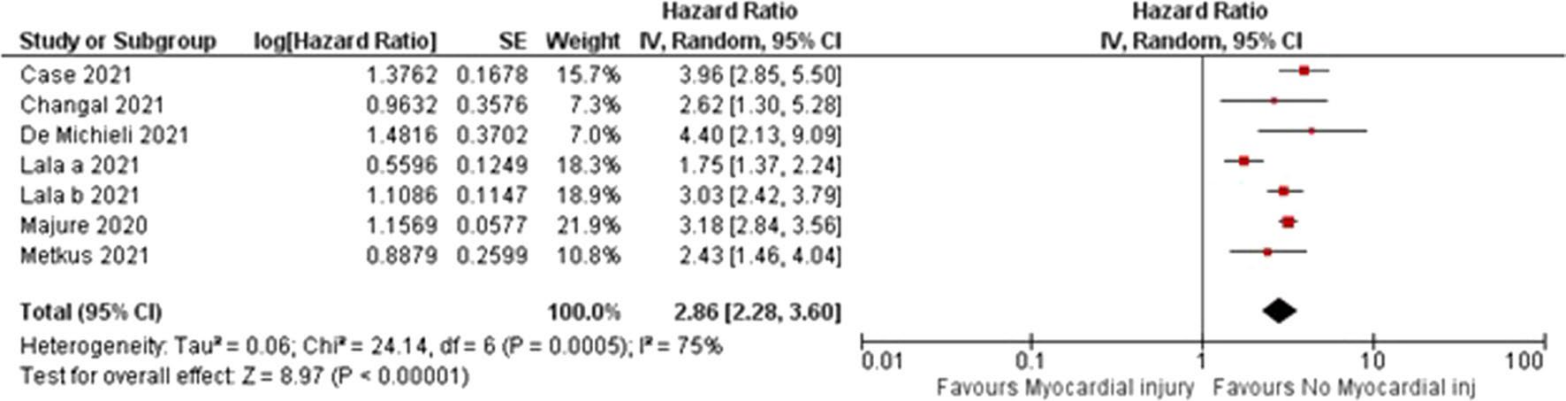
Forrest plot demonstrating Hazard ratio for mortality in patients with myocardial injury compared with no myocardial injury. Horizontal lines represent 95% confidence intervals (CI). The rectangles represent the point estimate, and the size of the rectangle is proportional to the weight given to each study in the meta-analysis. The diamond represents the summary estimate (size of the diamond = 95% CI). The vertical line represents the reference of no increased risk. The study by Lala et al. is divided into Lala a and Lala b. Lala a includes patients with troponin I > 0.03–0.09 ng/ml, Lala b includes patients with troponin I > 0.09 in the myocardial injury group

In the meta-analysis, we found the presence of myocardial injury was significantly associated with mortality (HR 2.43, CI 2.28–3.6, *P* 0.0005, Fig. 1). Each study found an increased risk of mortality with myocardial injury.

## Discussion

This study and review show myocardial injury is common (9–52%) in hospitalized COVID-19 patients in the U.S. [9–14]. This is in contrast to studies from China, which show relatively lower (7–28%) rates of myocardial injury in hospitalized COVID-19 patients [15–18]. We also found that development of myocardial injury in COVID-19 U.S. patients is associated with increased mortality, change of code status to “do not resuscitate”, discharge to skilled nursing facilities instead of home, development of acute kidney injury, circulatory shock, and arrhythmias.

We have, for the first time, shown in a meta-analysis on studies performed in the U.S. that myocardial injury significantly increases the mortality among hospitalized COVID-19 patients. This provides a high level of evidence. Similar to our results, a report of 416 patients from Wuhan, China demonstrated an HR of 3.41 (95% CI 1.62–7.16) for death in patients with myocardial injury compared with patients without myocardial injury [15]. Of note, *Nuzzi V *et al*.* found that in-hospital troponin elevation in Caucasian patients without myocardial injury at admission has a strong correlation with mortality [19]. We recommend that all patients admitted with COVID-19 should have troponin levels checked during their hospitalization. This, along with other clinical and laboratory variables, can provide further guidance on management and prognosis. The association of troponin elevation with increased mortality likely has two main reasons. Firstly, troponin elevation in COVID-19 is more likely to occur in patients with underlying cardiac and non-cardiac comorbidities, placing them at an increased risk of poor outcomes. Secondly, cardiac injury in the setting of COVID-19 is a marker of tissue hypoxia, myocardial cytotoxicity, systemic cytokine upregulation, demand–supply mismatch, thrombosis, and plaque vulnerability [20–22]. All of these processes suggest advanced disease and poor prognosis.

The underlying factors that predispose to the development of myocardial injury are age, hypertension, underlying CVDs, and chronic kidney disease. The CVDs associated with the development of myocardial injury are ischemic heart disease, heart failure (both preserved and reduced EF), and atrial fibrillation. Our review of other U.S. studies suggests similar risk factors for developing myocardial injury [9–14].

While some studies performed outside of the U.S. have investigated the association of myocardial injury with COVID-19, such studies have often used non-guideline definitions that utilize imaging and electrocardiographic abnormalities for establishing diagnosis. This increases the margin of error in diagnosing the patients with true myocardial injury, making such studies less reliable. We utilized a uniform and validated definition for Type 2 MI in an attempt to avoid this error.

Although the patients with myocardial injury had poor outcomes, there was no significant difference in the healthcare costs. This is because many patients in the myocardial injury group were discharged to skilled nursing facilities rather than home, and likely would result in a higher overall cost. No other study in the review has studied the healthcare costs outcome.

There are some limitations to our study. All findings are retrospective in design and are thus limited by this. Another limitation is that only patients who had troponin levels checked during their hospitalization were included. However, only a small number of patients were excluded for lack of serum troponin testing (n = 11). All of the studies included patients prior to when vaccination against COVID-19 was available. Additionally, none of the included studies have provided data on coronary angiography in the included patients. Nevertheless, on this final point, diagnosis of myocardial injury is based on clinical, EKG, laboratory, and imaging data and coronary angiography is often not required to make this diagnosis. While some authors [23] have suggested a racial predisposition to adverse outcomes from COVID-19, the present study could not study the effect of race on mortality outcomes due to sample size limitations. Such data was also not available for meta-analysis.

To conclude, myocardial injury is a common phenomenon in hospitalized COVID-19 patients in the U.S. Elevated troponin in this population predicts a poor outcome and higher risk of mortality.

## Supplementary Information

**Additional file 1.** Search strategy and PRISMA checklist for the Meta-analysis.

## Data Availability

Will be provided upon reasonable request. Please email Mujeeb A. Sheikh MD (smujeeb73@gmail.com) for requests.

## Abbreviations

ACEi: ACE inhibitor
ARBs: Angiotensin II receptor blocker
ARNI: Angiotensin receptor-neprilysin inhibitor
BBB: Bundle branch block (complete left or right)
BNP: Brain natriuretic peptide
CVD: Cardiovascular disease
CVVHD: Continuous venovenous hemodialysis
ESRD: End stage renal disease
HD: Hemodialysis
HFpEF: Heart failure with preserved ejection fraction
HFrEF: Heart failure with reduced ejection fraction
hs-cTnT: High sensitivity cardiac troponin-t
LOS: Length of stay
SNF: Skilled nursing facility
SPSS: Statistical package for the social sciences
U.S.: United States
VT: Ventricular tachycardia
